# Anti-inflammatory and antiplatelet effects of non-vitamin K antagonist oral anticoagulants in acute phase of ischemic stroke patients

**DOI:** 10.1186/s40169-017-0179-9

**Published:** 2018-01-12

**Authors:** Taizen Nakase, Junta Moroi, Tatsuya Ishikawa

**Affiliations:** 1 Department of Neurology, Research Institute for Brain and Blood Vessels-Akita, 6-10 Sensyu Kubota Machi, Akita, 010-0874 Japan; 2 Department of Surgical Neurology, Research Institute for Brain and Blood Vessels-Akita, Akita, Japan

**Keywords:** Thrombin, Factor Xa, Blood platelets, Inflammation, Stroke

## Abstract

**Background:**

Recently, non-vitamin K antagonist oral anticoagulants such as direct thrombin and direct factor Xa inhibitors have been prescribed for prevention of embolic stroke. While in Japan, argatroban, also a direct thrombin inhibitor, is available for the treatment of atherothrombotic stroke patients. This study aimed to explore whether there is any differences between direct thrombin and direct factor Xa inhibitors regarding the inhibiting effect against thrombogenesis in the clinical setting of acute ischemic stroke.

**Methods:**

Acute ischemic stroke patients newly prescribed anti-thrombotic agents were consecutively screened, and 44 patients with single medicine were enrolled (median 72.0 years-old). Blood samples were obtained at 1 and 2 weeks after the medication started. The extent of anticoagulation activity, inflammatory markers and platelet aggregation were assessed. Patients with antiplatelets were used as control.

**Results:**

Prescribed antithrombotics were dabigatran (group D: n = 12), apixaban (group A: n = 14) and antiplatelet agents (group P: n = 18). Prevalence of stroke risks and anticoagulation activity were not different between groups D and A. The alteration of inflammatory markers in a week in the group A showed similar trend to those in the group P. The group D presented relatively lower amount of high-sensitive C-reactive protein and higher amount of pentraxin-3 compared with groups A and P. While 88.9% of group P patients showed decreased platelet aggregation activity with adenosine diphosphate, 55.6% of group D and 40.0% of group A presented the inhibition of platelet aggregation activity.

**Conclusions:**

Even in acute ischemic stroke patients, both apixaban and dabigatran equally showed the anticoagulation activity. The reduction of inflammatory response might be prominent in apixaban, whereas the inhibition of platelet aggregation activity might be evident in dabigatran.

## Background

Recently non-vitamin K antagonist oral anticoagulants (NOACs), such as dabigatran, apixaban, rivaroxaban and edoxaban, have been used for preventing embolic stroke. The clinical trial data in NOACs reported the lower risk of hemorrhagic complication compared with warfarin [1–4]. Therefore, it can be said that it is preferable to use NOACs for preventing ischemic stroke in patients with high hemorrhagic risks [5]. Moreover, NOACs have recently been reported to show not only anticoagulation effect but also anti-inflammatory and antiplatelet effects [6–9]. In Japan, actually, argatroban which is also a direct thrombin inhibitor is available for treating acute atherothrombotic stroke patients [10, 11], supported by the evidence of decreasing micro thrombus from the fragile atheromatous plaques [12]. Meanwhile, other studies reported that NOACs did not present any effect against inflammatory response nor platelet activation [13–16]. It can be said that the pleiotropic effect of NOACs are still under debate. Therefore, if the anti-inflammatory effect and antiplatelet effect of NOACs will be revealed, it may influence on the decision making of prescription of antithrombotic agents including combination use of antiplatelet and anticoagulation medicines.

This study targeted acute ischemic stroke (AIS) patients, because the inflammatory response and platelet activity would be amplified in this condition. Then, we investigated whether there is any difference in these pleiotropic effects of NOACs along with anticoagulation effect or not, especially between direct thrombin inhibitor and direct factor Xa inhibitor.

## Methods

### Patients

**Fig. 1:**
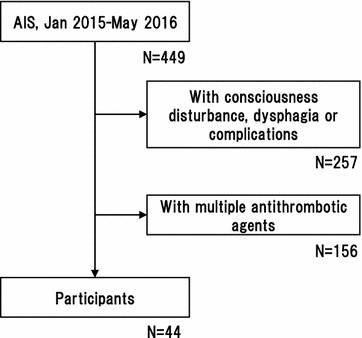
The inclusion and exclusion criteria with number of patients

**Fig. 2:**
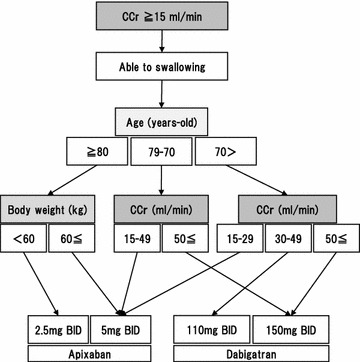
The algorithm of selection of non-vitamin K antagonist oral anticoagulant (NOAC). This arm is used when a patient prefer a medicine twice a day administration. A patient with poor kidney function (CCr < 15 ml/min) cannot apply this algorithm. If a patient shows dysphagia, a NOAC which can be pulverized is considered for prescription. *CCr* creatinine clearance, *BID* twice a day oral administration

All procedures in this study was approved by the ethical committee of the Research Institute for Brain and Blood Vessels-Akita (#14-7). Between January 2015 and May 2016, AIS patients newly prescribed antithrombotic agents were consecutively screened, and 44 patients were enrolled (30 male and 14 female, median age 72.0 years-old). All patients were consented with written document. Patients who had multiple antithrombotic agents, consciousness disturbance, dysphagia and complications such as pneumonia or active malignancy were excluded (Fig. 1). Thus far, stroke severity became similar level among the enrolled patients. Blood sampling was performed at 1 and 2 weeks after the antithrombotic medication became a single kind. Evaluated laboratory data were activated partial thromboplastin time (APTT), prothrombin time (PT), the amounts of prothrombin fragment (F1 + 2), interleukin-6 (IL-6), high sensitivity C-reactive protein (hsCRP) and pentraxin-3 (PTX3). The activity of platelet aggregation elicited by adenosine diphosphate (ADP) or collagen was evaluated using an aggregation analyzer (PA-200, Kowa Co. Ltd., Tokyo, Japan). The anti-inflammatory and antiplatelet effects were compared between patients with dabigatran and apixaban. Patients with antiplatelets were used as control. The selection of prescribing antithrombotic medicines was basically followed by the guidelines [17, 18]. Especially for the choice of NOACs, the algorithm in our hospital was used (Fig. 2). The initiation of antithrombotic agents was at the appropriate days after stroke onset following the guideline [19]. The average day was at 9.6th day (range: 1–18 days) in dabigatran, at 12.8th day (range: 1–30 days) in apixaban and at 10.5th day (range: 1–20 days) in antiplatelets.

Background characteristics of each patient were collected from patient’s clinical records. Stroke subtype was classified into (1) cardioembolic stroke, (2) large artery atherothrombotic infarction and (3) small vessel occlusion based on the criteria of the Trial of Org 10172 in the acute stroke treatment classification system [20]. Risk factors were defined as hypertension (HT), hyperlipidemia (HL), diabetes mellitus (DM) and smoking.

### Statistical analysis

All data are presented as mean ± standard deviation (SD) for continuous variables and a number and percentage (%) for categorical variables. Patients’ characteristics were compared with Pearson’s χ^2^ test among different groups. Comparisons of the amount of inflammatory markers were performed with Kruskal–Wallis one-way analysis of variance. All statistical analysis was performed by JMP9 software (SAS Inst. Inc. Cary, NC).

## Results

### Patients’ background characteristics

Prescribed antithrombotics were dabigatran (group D: n = 12), apixaban (group A: n = 14) and antiplatelet agents (group P: clopidogrel n = 8 and cilostazol n = 10). There was no significant difference of age, sex distribution and atherosclerotic risk factors, such as hypertension, dyslipidemia and diabetes mellitus among three groups. As known that the anticoagulants are prescribed to embolic stroke patients, the frequency of atrial fibrillation was higher in the group D and A compared with the group P (Table 1).

**Table 1:** Patients’ characteristics

	Total	Group D	Group A	Group P	P value
N	44	12	14	18	
Sex (m/f)	30/14	9/3	9/5	12/6	0.829
Age (mean ± SD)	70.8 ± 11.8	69.6 ± 14.3	74.1 ± 12.1	69.1 ± 9.8	0.335
(median)	72.0	69.5	80.0	70.5	
Hypertension	68.2%	66.7%	50%	83.3%	0.132
Dyslipidemia	29.5%	8.3%	28.6%	44.4%	0.104
Diabetes	38.6%	33.3%	21.4%	55.6%	0.131
Atrial fibrillation	34.1%	58.3%	57.1%	0%	< 0.001
Smoking	54.5%	66.7%	57.1%	44.4%	0.475
Stroke subtype (n)					< 0.001
CE	23	9	14	0	
LAA	10	2	0	8	
SVO	11	1	0	10	

*CE* cardioembolism, *LAA* large artery atherothrombosis, *SAO* small vessel occlusion

Laboratory data was shown in Table 2. APTT was prolonged only in the group D. Whereas, the groups A and P showed the same amount of APTT as the normal range. Both groups D and A showed the extended PT and decreased F1 + 2 amount.

**Table 2:** Anticoagulation indicators

	Group D		Group A		Group P	
	1 week	2 week	1 week	2 week	1 week	2 week
APTT (24–34 s)	47.3 ± 9.7	50.1 ± 12.2	33.6 ± 4.9	31.8 ± 5.4	29.8 ± 2.3	30.0 ± 2.2
PT (11–14 s)	16.4 ± 1.9	16.3 ± 1.8	15.4 ± 2.6	15.0 ± 2.3	13.1 ± 0.5	13.0 ± 0.6
F1 + 2 (69–229 pmol/l)	150.4 ± 62.3	159.0 ± 62.1	130.9 ± 40.5	182.0 ± 116.6	230.7 ± 59.9	247.2 ± 76.9
IL-6 (< 8 pg/ml)	2.9 ± 4.2	2.3 ± 1.4	5.1 ± 4.8	3.1 ± 1.9	5.3 ± 6.7	2.4 ± 2.5
hsCRP (< 0.30 mg/dl)	0.10 ± 0.14	0.11 ± 0.13	0.22 ± 0.21	0.20 ± 0.19	0.19 ± 0.20	0.12 ± 0.16
PTX3 (0.73–5.49 ng/ml)	3.4 ± 3.8	2.9 ± 2.4	3.9 ± 2.8	3.5 ± 2.1	3.3 ± 2.5	3.0 ± 2.3

Normal range of each factor is indicated in parentheses. Data are presented as average ± standard deviation

*APTT* activated partial thromboplastin time, *PT* prothrombin time, *F1* *+* *2* prothrombin fragment, *IL-6* interleukin 6, *hsCRP* high sensitivity C-reactive protein, *PTX3* pentraxin 3

### The anti-inflammatory effect

As shown in Table 2, IL-6 was slightly lower in the group D compared with the groups A and P at the 1st week (2.9 ± 4.2, 5.1 ± 4.8 and 5.3 ± 6.7 ng/ml, respectively. Not significant), then the amounts became the same level among all three groups at the 2nd week (2.3 ± 1.4, 3.1 ± 1.9 and 2.4 ± 2.5 ng/ml, respectively). The amount of hsCRP was lower in the group D compared with groups A and P at the 1st week (0.10 ± 0.14, 0.22 ± 0.21 and 0.19 ± 0.20 mg/dl, respectively. Not significant). While the group P showed the decline of hsCRP in the 2nd week (0.12 ± 0.16 mg/dl), the groups D and A showed an equivocal level in the 2nd week (0.11 ± 0.13 and 0.20 ± 0.19 mg/dl, respectively). The amount of PTX3 exhibited similar level among all three groups both at the 1st and 2nd weeks, and there was no significance.

### The antiplatelet effect

**Fig. 3:**
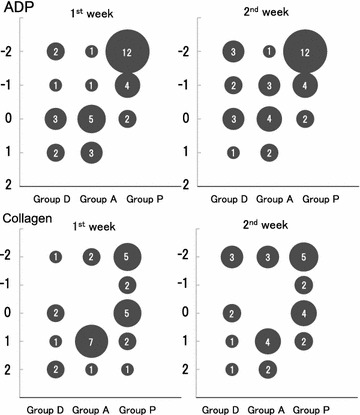
Platelet aggregation activity elicited by ADP or collagen. The number of patients in whom platelet aggregation activity is reduced are increasing in 1 week in both groups D and A. This finding is observed in the same tendency as for elicitation by ADP and collagen. Bubble size and the number indicate the number of patients. Y axis expresses the degree of platelet coagulation activity. Negative number indicates reduced activity and positive number indicates accelerated activity

Figure 3 showed the bubble chart graph of platelet aggregation activity. Four of 12 patients in the group D and 4 of 14 patients in the group A were excluded from the analysis because of the insufficient data of platelet aggregation activity. As expected in the group P, 16 of 18 patients (88.9%) showed the reduced platelet aggregation activity with ADP at both 1st and 2nd weeks. Two patients remained in the equivocal level of platelet aggregation. Whereas, 7 of 15 patients (46.7%) at the 1st week and 7 of 13 patients (53.8%) at the 2nd week showed the reduced platelet aggregation activity with collagen. In the group D, 3 of 8 patients (37.5%) at the 1st week and 5 of 8 patients (55.6%) at the 2nd week showed the reduced platelet aggregation activity with ADP. Moreover, 1 of 6 patients (16.7%) and 3 of 7 patients (42.9%) presented the inhibition of platelet aggregation activity with collagen at the 1st and 2nd weeks, respectively. In the group A, 2 of 10 patients (20.0%) at the 1st week and 4 of 10 patients (40.0%) at the 2nd week showed the reduced platelet aggregation activity with ADP. While, 2 of 10 patients (20.0%) and 3 of 9 patients (33.3%) presented the reduced platelet aggregation activity with collagen at the 1st week and 2nd week, respectively.

During the 1 month observation period, no patients had stroke recurrence or hemorrhagic complication.

## Discussion

This study clearly demonstrated that, even in the acute ischemic stroke patients, both dabigatran and apixaban could express not only anticoagulation effect but also some amount of anti-inflammatory and antiplatelet effects.

Regarding the indicator of anticoagulation effect, APTT was reported to be prolonged not by apixaban but by dabigatran [21, 22], supported our findings. Moreover, according to the observed data of PT and F1 + 2, it can be said that both dabigatran and apixaban might accurately be able to express the anticoagulation activity in the acute phase of ischemic stroke.

Along with the activation of fibrinogen, thrombin can effect on platelet membrane and cleave protease activated receptor (PAR)-1 and PAR-4, partly participating in the platelet aggregation [23, 24]. Moreover, in normal condition, thrombin combined with thrombomoduline can activate the protein C at the vascular endothelia. Then, the activated protein C (APC) provides not only anticoagulation effect but also anti-inflammatory effect [25, 26]. However at the advanced atherosclerotic lesion, the amount of APC might be declined, and the effect of thrombin may become dominant [27]. Therefore, among the patient of acute ischemic stroke or with advanced atherosclerotic lesion, thrombin might play an accelerative role in inflammation and coagulation.

This study showed that dabigatran might decrease the inflammatory response by means of reducing IL-6 and hsCRP within 1 week under the stimulated condition. In fact, dabigatran was reported to reduce the formation of atheromatous thrombus by decreasing the inflammatory response in ApoE knock-out mice [28]. Apixaban was also reported it’s anti-inflammatory effect by reducing the production of free radicals in in vitro ischemic stress model [8]. In this study, the amount of IL-6 and PTX3 in apixaban presented the same trend as those in antiplatelets. Since PTX3 is reported to relate to vascular inflammation [29], it can be said that apixaban might show anti-inflammatory effect like antiplatelet agents [30, 31]. Very recent study reported that antithrombotic therapy with dabigatran plus antiplatelet medicine showed significantly lower risk of hemorrhagic complication compared with triple therapy (warfarin plus dual antiplatelet medicines), along with no difference of the risk of thromboembolic events between two therapies [32]. Considering the findings of our study, if dabigatran will be prescribed as anticoagulation agent, it may replace warfarin plus single antiplatelet agent, and we might be able to avoid triple therapy (anticoagulation plus dual antiplatelets).

According to previous reports, the platelet aggregation was normally observed in healthy volunteer taking dabigatran or rivaroxaban [16]. The platelet aggregation induced by ADP was reported not to be changed in blood samples obtained from chronic atrial fibrillation patients by taking dabigatran and warfarin [14]. While, apixaban was reported to show the inhibitory effect of platelet aggregation in vitro [15]. Herein, this study focused on acute ischemic stroke patients in whom the platelet aggregation might be elicited. Then, both dabigatran and apixaban were observed to express some amount of antiplatelet effect under the stimulated condition. Actually, our findings can be supported by other studies in which argatroban, another direct thrombin antagonist, could inhibit the platelet aggregation [33] and decrease the amount of microemboli from unstable atherothrombotic plaque [12, 34].

There are some limitations in this study. First, the number of sample cases was small, and the statistical analysis was not able to evaluate enough. Even though, we screened patients who took the only one antithrombotic agent during the study period, so that the data should not be contaminated by any other medicines. Second, this study adopted patients with antiplatelet medicines as control. Since prescribed antithrombotic agents were various depend on stroke subtype, it is not enough to compare the anti-inflammatory and antiplatelet effects among patients prescribed NOACs or antiplatelets. Moreover, it was quite difficult to set control group in which no antithrombotic agent was prescribed, since this study was conducted under standard stroke treatments. Nevertheless, this is the first report in which the pleiotropic effects of dabigatran and apixaban were assessed in the acute ischemic stroke patients. A large scale prospective study will be needed to confirm our current findings.

## Conclusions

Even in the setting of acute ischemic stroke, the effect of anticoagulation activity was equally expressed in dabigatran and apixaban. The anti-inflammatory effect might be observed in apixaban as the same trend as that in antiplatelet agents. Whereas, the antiplatelet aggregation effect might be stronger in dabigatran compared with that in apixaban.

## Abbreviations

AIS: acute ischemic stroke
APTT: activated partial thromboplastin time
PT: prothrombin time
F1 + 2: the amounts of prothrombin fragment
IL-6: interleukin-6
hsCRP: high sensitivity C-reactive protein
PTX3: pentraxin-3
ADP: adenosine diphosphate
NOAC: non-vitamin K antagonist oral anticoagulant
HT: hypertension
HL: hyperlipidemia
DM: diabetes mellitus
SD: standard deviation
APC: activated protein C
PAR: protease activated receptor
